# How health system factors affect primary care practitioners’ decisions to refer patients for further investigation: protocol for a pan-European ecological study

**DOI:** 10.1186/s12913-018-3170-2

**Published:** 2018-05-08

**Authors:** Michael Harris, Gordon Taylor, The Örenäs Research Group

**Affiliations:** Department for Health, University of Bath, Claverton Down, Bath, BA2 7AY UK

**Keywords:** Delivery of health care, Primary health care, Cancer, Decision making, Diagnosis, Survival

## Abstract

**Background:**

There is wide variation in the overall one-year relative cancer survival rates across Europe, and this is thought to indicate national variations in stage of disease at diagnosis. However, there is little evidence to explain how different national systems influence a primary care practitioner’s (PCP’s) referral decisions, and how these relate to the variation in survival rates.

This study investigates the health system factors that influence the thinking of PCPs when faced with patients who may have cancer, how they compare across European countries, and how they relate to national one-year relative cancer relative survival rates.

**Methods:**

An online quantitative questionnaire with closed-ended questions is used in a cross-sectional survey of 1250 PCPs in Europe, in 25 local health areas in 20 countries. Descriptive data are elicited for each country, including respondents’ demographic details.

An exploratory factor analysis will identify factors underlying the decision to refer patients for further investigations. Between-country variation in these factors will then be further investigated and presented as means with 95% confidence intervals. A regression model will be fitted for the vignettes using one-year relative survival as the outcome, with the proportion of PCPs opting to investigate as a single explanatory variable. Weighted regression will be used to explore which health system factors are associated with opting to investigate and with one-year relative survival.

Linear correlations will be estimated between the proportions opting to investigate and national survival rates. When comparing between countries, weighted linear regression will be used to adjust for different sample sizes in each country.

**Discussion:**

This study investigates which system factors affect PCPs’ decisions to refer and investigate patients who may have cancer, how they compare across 20 European countries, and how these factors relate to cancer survival rates.

Knowledge of the extent and variability of the health system factors that affect referral decisions will inform future health service design, policy and research.

## Background

### Variation in European survival rates

There is wide variation in the relative cancer survival rates across Europe [1]. For example, in the United Kingdom, more than 6000 deaths within five years of diagnosis would have been avoided annually if survival in Britain had matched the European mean [2], representing 6-7% of deaths due to cancer. The European 1-year relative cancer survival rates vary even more than those for 5-year survival: analysis of the EUROCARE-5 data shows that the 1-year relative survival rate for all cancer sites varies from 60.0 to 80.5% between European countries, and there is with large variation even within EUROCARE’s five main European regions [1]. One-year relative cancer survival rates may be affected by differences in registration (e.g. completeness and use of death certificates), and lead-time and over-diagnosis biases [3, 4], however they are commonly thought to be a marker of more advanced disease at diagnosis [5, 6].

Analysis of European one-year relative cancer survival results [1, 7–9] shows that some countries have high survival rates for most cancers (including Belgium, France, Sweden and Switzerland), while others have lower survival rates (including Bulgaria, Croatia, Poland and Scotland). Recent cancer survival rates have shown improvement [9], but the between-country survival differences have shown little change [10]. Survival variation in the subsequent four-year period is narrower, and this suggests that an improvement in cancer awareness and early detection in relatively poorly performing countries could reduce the observed one-year relative survival gap [8].

### Consequences of late diagnosis

The assumption that earlier diagnosis of cancer in symptomatic patients leads to improved survival is difficult to confirm [11]. A systematic review found that, while the survival benefits of earlier diagnosis varies between cancers, there is an association between a shorter diagnostic interval and improved outcomes for breast, colorectal, head and neck, and testicular cancers, as well as for melanoma [12]. In addition, a study of data for breast, colorectal and lung cancer in England determined that late diagnosis was highly likely to be a major factor explaining poorer survival in those cancers [5].

There are considerable difficulties in deciding how to achieve more timely cancer diagnoses [13]. A Primary Care Practitioner (PCP) will see only a small number of new cancers in any one year (309,500 UK patients were diagnosed with cancer in 2008 [14], giving a mean of 7.5 per PCP), and a General Practitioner (GP) may rarely or never see some uncommon cancers. In most patients who present with cancer-related symptoms those symptoms are undifferentiated, which makes them less likely to be initially interpreted being due to cancer [13].

### Access to specialist care and investigations

Healthcare systems with strong primary care have lower mortality and morbidity rates [15], and GP gatekeeping is the cornerstone of many European medical systems [16]. There has been some evidence that countries with gatekeeper systems have lower one-year relative cancer survival than those that do not have GPs as gatekeepers [17]. This may be because gatekeeping systems can impose cost and resource decisions which impede early referral for investigation [18]. Gatekeeping may act against early cancer diagnosis by changing the balance between referral for opinion or investigation, and minimising costs and the risk of iatrogenic illness [19].

However, a European study found large national differences in the extent of gatekeeping, with no uniform “GP-as-gatekeeper” model, and no link between a higher probability of initial consultation with a GP and reduced cancer survival [20].

There is little evidence on how different healthcare systems influence a primary care practitioner’s referral decisions, or on how they may contribute to the marked variations in one-year cancer survival [13].

### The effect of different beliefs, health systems and behaviour in primary care

The International Cancer Benchmarking Partnership (ICBP) is using predominantly quantitative methods to measure beliefs, behaviours and systems in primary care as they relate to delays in diagnosis [21]. The ICBP’s work has examined the differences in cancer awareness and beliefs between six countries with comparable wealth to detect where these might help to explain to those countries’ survival patterns [22].

### Study rationale and evidence gap

In order to improve outcomes for patients, a better understanding of how individual health system factors and patient and professional behaviour interact is needed [23]. One possible explanation of differences in survival between countries is differing levels of diagnostic activity, and there is a need for studies that fully interpret the possible reasons for differences in cancer survival and suggest how to remedy disparities [9].

A recently published ICBP study showed a correlation between cancer survival rates and how ready PCPs are to investigate symptoms that could be due to cancer [24]. While the study found no correlation between some health system factors and readiness to refer, there was no exploration of how individual doctors felt that those system factors affected their decision-making. However, a large variety of non-clinical factors that are likely to affect PCPs’ referral decisions have been identified [25]. These include the extent to which PCPs are gatekeepers, funding systems, access to special investigations, concerns over the risk of litigation, and the barriers to accessing specialist opinions.

To develop a more detailed understanding of these issues, this study investigates which system factors affect PCPs’ decisions to refer patients who may have cancer for further investigation (specialist opinion or special investigation) in twenty European countries, how they compare across those countries, and how those factors relate to one-year relative cancer survival rates.

Knowledge of the extent and variability of the health system factors that affect referral decisions will inform future health service design, policy and research.

## Methods and design

The study uses a questionnaire survey of PCPs in twenty European countries with diverse cancer survival rates. The questionnaire was administered online. Recruitment started in November 2015 and was completed at the end of 2016.

### Study population

The Örenäs Research Group is a pan-European collaborative of primary care researchers, formed in 2013 to study the factors influencing national variations in the early diagnosis of cancer in primary care. The research is being conducted in 25 Örenäs Research Group centres in 20 countries across Europe: Bulgaria, Croatia, Denmark, England, Finland, France, Germany, Greece, Israel, Italy, Netherlands, Norway, Poland, Portugal, Romania, Scotland, Slovenia, Spain, Sweden and Switzerland. Medical doctors were eligible for the survey if they were doctors working mainly in primary care. These doctors, here referred to collectively as ‘Primary Care Practitioners’, included GPs and other doctors who had had specialist training but worked in the community and could be accessed directly by patients without referral.

### Sampling methods

Each local lead was asked to email an invitation to take part in the survey to all the PCPs in their local health district, and to recruit at least fifty participants. Low survey response rates are common in primary care [26] and can vary between jurisdictions, with a recent ICBP survey response rate varying from 5.5 to 45.6% depending on country [24]. Any local leads who had difficulty in achieving the required sample sizes were therefore asked to increase the number of responses by using snowballing, a recognised technique for recruiting hard-to-reach populations in health studies [27, 28].

### Sample size

A total sample size of 1000 or more responses was calculated to be sufficient to obtain stable factor estimates within the exploratory factor analysis [29]. Given the 20 items in the questionnaire, this is also considerably in excess of the Hair et al. (1998) rule of requiring a sample size of at least ten times the number of variables [30]. Based on a minimum of 50 responses for each jurisdiction this will provide us with 95% confidence intervals (CI) ±14% for equally distributed responses, and 95% CI ±13% for less equally distributed responses, enabling informal exploratory comparisons between jurisdictions.

### Exploratory work: results and study adaptation

The study was designed by Örenäs Research Group investigators at a meeting in May 2014. They developed and agreed by consensus a list of factors that may affect a PCP’s decisions to refer patients for further investigation. A literature review did not reveal any missing factors. A questionnaire with five clinical vignettes (two of which were adapted, with permission, from ICBP vignettes) and 45 decision-making factors was piloted by the Örenäs Research Group local leads in January 2015 to check validity. One of the vignettes and six of the factors were found to be invalid and were removed.

A questionnaire, in English, with the remaining 39 decision-making statements was piloted by 49 PCPs in 16 Örenäs Research Group member countries in July 2015, to identify the statements on which there was little or no difference in responses between countries. Of those, 19 statements were found to show little or no variation between countries so were removed from the questionnaire, leaving 20 statements.

Örenäs Research Group leads arranged for translations of the questionnaire into their local languages where these were not English, a total of 19 translations from the original English. Translation and validation were done in a standardised way [31]: medically qualified native speakers of the local languages who were fluent in English did the ‘forward’ translations. ‘Backward’ translations into English were then made by translators who were fluent in both English and their local languages. The forward translations were then compared with the backward ones, to assess semantic and conceptual equivalence [32]. Discrepancies between the forward and backward translations were resolved by discussion with the translators, following which the final translations were agreed on. The validated questionnaires were then put on-line by MH using the survey tool provided by SurveyMonkey, and each national lead was issued with their own web link. Because of the widespread geographical coverage, on-line methodology was used to aid the logistics of survey administration; on-line surveys have been successfully used in healthcare-professional research [33].

Local Örenäs Research Group leads each then sent their survey link and a translated participant information sheet to local PCPs, including doctors who had had specialist training but worked in the community and could be accessed directly by patients without referral. Consent was implied by agreeing to take part in the survey.

As well as managing recruitment of participants in their respective countries, Örenäs Research Group local leads will contribute to the interpretation of the results and their dissemination.

### Description of the questionnaire

The questionnaire is made up of 47 items and divided into four sections:

A. Demographic questions: five questions ask about years since graduation, gender, speciality, type and rural/urban location of practice and number of doctors working in practice.
B. Referral availability questions: two questions ask about tests and specialist opinions that are either directly or indirectly available to the respondent.
C. Clinical vignette questions: this section describes four patients who have symptoms suggestive of lung, colorectal, ovarian and breast cancer respectively; for each patient, a range of five possible management decisions (including whether the respondent would investigate or refer the patient, or would use watchful waiting) is given, with a ‘yes/no’ option for each.
D. Health system factor questions: twenty statements relating to health system factors that may affect referral decisions are given, with a request for the respondent to state how much they agree with each statement. A five-point Likert rating scale is provided for participants, with response options ranging from ‘Strongly agree’ to ‘Strongly disagree’.

The health system factor statements are divided into seven sections:

1. Guidelines and local systems (example: ‘The local health system encourages us to refer any patients with possible cancer early, even if there is a low risk of cancer’).
2. Relationship with specialist colleagues (example: ‘Here, specialists usually welcome referrals).
3. Financial issues (example: ‘Here, high quality care for an individual patient is always more important than costs’).
4. Concerns about complaints (example: ‘My colleagues sometimes criticise me if I have referred a patient to them, but they think that I should have been able to manage the patient myself’).
5. Waiting lists (example: ‘We have access to a fast-track specialist appointment system for patients with suspected cancer’).
6. The effect of PCPs’ workload (example: ‘I am usually very busy, so I sometimes refer to help reduce my workload’).
7. Patient and doctor feelings (example: ‘I am likely to refer if the patient says that she/he would like to be referred, even if there are no “red flags”’).

### Statistical analysis

Descriptive data will be provided on each country, including the number and characteristics of the PCPs completing the survey.

An exploratory factor analysis will be undertaken on the questionnaire responses, to identify underlying factors relating to the decision to refer patients for further investigations. We will use a principal components method [34], with a direct oblimin rotation to allow for correlated factors. The number of components will be defined by inspection of the scree plot and the Kaiser criterion (eigenvalue ≥1). Between-country variation in these factors will then be further investigated and presented as means with 95% confidence intervals. A regression model will be fitted for the vignettes using overall national one-year relative cancer survival, calculated from EUROCARE data [1], as the outcome, with the proportion of PCPs opting to investigate as a single explanatory variable. Weighted regression will be used to explore which health system factors are associated with opting to investigate and with one-year relative survival. The primary outcome measure will be one-year relative cancer survival, as it is thought to reflect activity in primary care. We will make a sensitivity analysis using five-year relative cancer survival.

We will estimate the linear correlations between the proportions of PCPs opting to investigate and national survival rates. For national comparisons, weighted linear regression will be used to adjust for different sample sizes in each country.

## Discussion

This research will consider how Europe’s differing models of primary care result in differences in the thresholds that PCPs have for considering a diagnosis of cancer and for investigating that possibility. By understanding the key differences and similarities between European countries with different cancer survival rates, we will be able to offer suggestions on how improvements can be made. The research findings will help all European countries improve their cancer survival rates by optimising cancer diagnosis policies and services.

### Strengths

To our knowledge, this is the first study that has been designed to examine the factors underlying PCPs’ referral decision-making, and to provide international comparisons of the extent to which PCPs themselves perceive these as important.

There are participating centres in four countries from each of the Central, Eastern, Northern, Southern and Western European geographical areas, providing variation in geography, health systems and levels of healthcare spending. It includes the views of PCPs who are not usually involved in research. The questionnaire was carefully developed and piloted by GPs and other PCPs, and therefore grounded in their clinical experience.

### Limitations

Collaborators are asked to recruit local primary care doctors by convenience sampling, and there may be selection bias [35]. The recruitment method used in this study (an emailed, unreimbursed survey, with a confidential approach that precludes follow-up of non-responders) may result in low response rates, leading to non-response bias and loss of power [26]. The survey has 47 items, which may cause response fatigue, and there may be bias due to hypothesis guessing [36]. There may also be a response bias, including social desirability bias [37], in participants’ answers to the vignettes and health system factor questions. While Likert scales are likely to be easily understood by participants and the responses are easily quantifiable, only five response options are given in the questionnaire, and these may fail to measure respondents’ true attitudes. Participants’ responses may be influenced by previous questions, and there may be country-level differences in response styles, for instance choosing or avoiding the ‘extreme’ options on the scale. [38].

Vignette-based methodologies are frequently used to examine judgments and decision-making processes, and here are used as proxies for real patients, but the vignettes may not be typical of patients seen by participant PCPs in their everyday practice. Despite this potential limitation, well-designed vignette studies that ask questions about judgments and decision-making can be highly generalisable to ‘real-life’ behaviour [39, 40], while avoiding the ethical and practical restrictions associated with other methods.

While EUROCARE-5 provides survival data for almost nine million adults in 29 countries, 8 of these countries have only partial coverage [9], and this may affect the generalizability of their data. One-year relative survival rates for cancer can be affected by lead-time and over-diagnosis biases [3, 4]. Although we will make a sensitivity analysis using five-year relative survival, this may not completely negate the risk of these biases.

### Expected impact

The study should indicate how much variation there is in PCP decision-making in participant countries, and how much of this variation can be explained by differences between the health systems. The results will indicate the policy domains where countries might be able to modify their systems to better support their GPs and other PCPs in the timely referral and investigation of patients who could have cancer.

The Örenäs Research Group will be able to compare the factor analysis scores with national cancer outcomes. These outcomes could include mortality, stage distribution and patient evaluations. An additional area of study could be to relate the factors and scores to national health system costs.

### Quality assurance

During the preparation of the study, quality has been ensured through the process of translation and back-translation of the questionnaire described above. The local leads for each participating centre proofread the translated documents to confirm that they reflected the terminology used in their health regions. The survey was then piloted by PCPs in each centre to check for clarity, formatting errors and cultural equivalence.

The Örenäs Research Group local leads share all decisions about research developments and outputs, with decisions made by consensus where possible, and by majority vote where this is not possible.

### Confidentiality

No personally identifiable information is collected from participants: the survey is anonymous.

### Dissemination

Sharing information about the project will take place throughout the duration of the work and not just at the end. Results will be published in peer-reviewed scientific journals as well as by conference presentations and will be communicated to respondents and relevant institutions through cooperation with the European General Practice Research Network. Local leads will be encouraged to publicise the project findings within their universities and health services, for example in newsletters, websites, meetings and local journal publications.

## Abbreviations

GP: General Practitioner
ICBP: International Cancer Benchmarking Partnership
PCP: Primary Care Practitioner
